# Pericardial Effusion/Cardiac Tamponade Induced by Peripherally Inserted Central Catheters in Very Low Birth Weight Infants: A Case Report and Literature Review

**DOI:** 10.3389/fped.2020.00235

**Published:** 2020-05-15

**Authors:** Ana Hou, Jianhua Fu

**Affiliations:** Department of Pediatrics, Shengjing Hospital of China Medical University, Shenyang, China

**Keywords:** Peripherally inserted central catheter, pericardial effusion, cardiac tamponade, very low birth weight, complication

## Abstract

**Background:** Although pericardial effusion/cardiac tamponade (PCE/CT) is a rare complication of peripherally inserted central catheters (PICCs), with an incidence of 0. 07–2%, it is associated with high mortality and is often life threatening. We sought to improve understanding of PICC-induced PCE/CT among pediatricians.

**Case presentation:** The clinical data of PICC-associated PCE/CT in a very low birth weight (VLBW) infant were summarized, and the relevant literature was also reviewed.

**Conclusions:** In VLBW infants with a PICC, if unstable respiratory circulatory system states are observed that cannot be explained, such as tachycardia, bradycardia, apnea, hypotension, and metabolic acidosis, PICC-induced PCE/CT should be considered. Early diagnosis and pericardial puncture are key to saving infants' lives.

## What Is Already Known on This Topic?

1. Pericardial effusion/cardiac tamponade (PCE/CT) is a rare complication.
2. PCE/CT has a high mortality and is often life-threatening.

## What This Paper Adds?

1. The catheter tip should be in the vena cava, but outside of the heart outline.
2. Catheter distortion is one of the reasons for the occurrence of PCE/CT.
3. Pericardiocentesis can significantly reduce the mortality of the disease.

## Background

Peripherally inserted central venous catheters (PICCs) can be retained for several weeks to months to avoid repeated venipuncture and also allow for infusion of high-concentration nutrient solution. PICCs are an important approach in rescuing very low birth weight (VLBW) infants and extremely low birth weight (ELBW) infants in neonatal intensive care units (NICUs), but they may cause severe complications, including catheter-related bloodstream infections, pleural effusions, arrhythmias, pericardial effusions (PCEs), and cardiac tamponade (CT) caused by PCE (1). The incidence of PCE/CT is 0.07–2% (2), but the mortality rate is as high as 75% without pericardiocentesis and 8% with pericardiocentesis (1). Therefore, PCE/CT is a very rare but life-threatening complication. Here, to improve clinicians' understanding of the disease, we report a case of PICC-induced PCE/CT in a VLBW infant in our department, review the relevant literature, summarize the clinical manifestations, and analyze the risk factors, including the position of the catheter tip.

## Case Presentation

### Case Report

**Figure 1:**
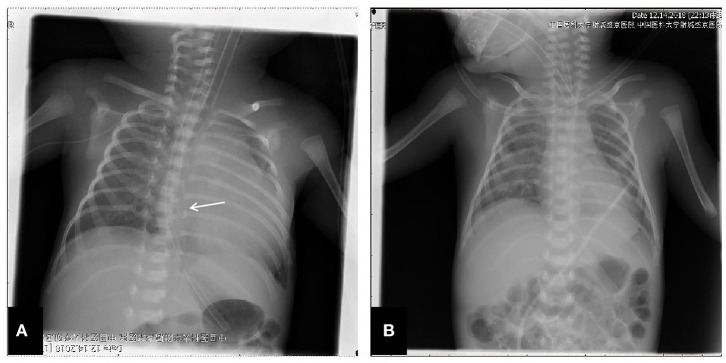
Chest X-ray presentation. **(A)** Chest X-ray performed at the 8th day after birth, which showed the distorted catheter as well as enlarged heart shadow; **(B)** Chest X-ray performed after the removal of PICC and the performance of pericardiocentesis, and the heart shadows were greatly reduced.

**Figure 2:**
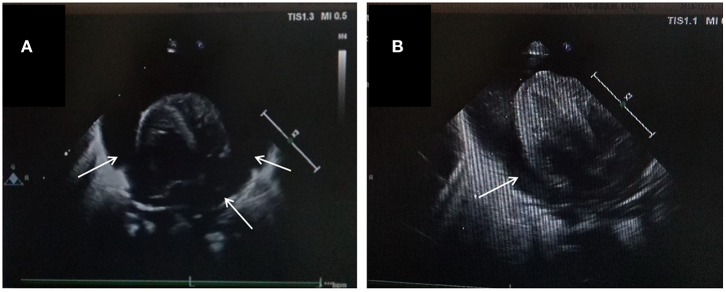
Echocardiographic image in the apical four chamber view shows a large pleural effusion. Pericardial cavity fluid dark area were detected: 6.2 mm in the rear of posterior wall of left ventricle, 6.5 mm in front of anterior wall of right ventricle, 9.1 mm in front of apical part, 5.6 mm outside the lateral wall of left ventricle, 9.8mm outside the lateral wall of right ventricle, 6.3 mm under the inferior wall of right ventricle beneath xiphoid process. Light spot reflex were visible in pericardial cavity. Left ventricular ejection fraction was 53%. **(A)** Apex four-chamber view of the heart. **(B)** Parasternal four-chamber view of the heart.

**Figure 3:**
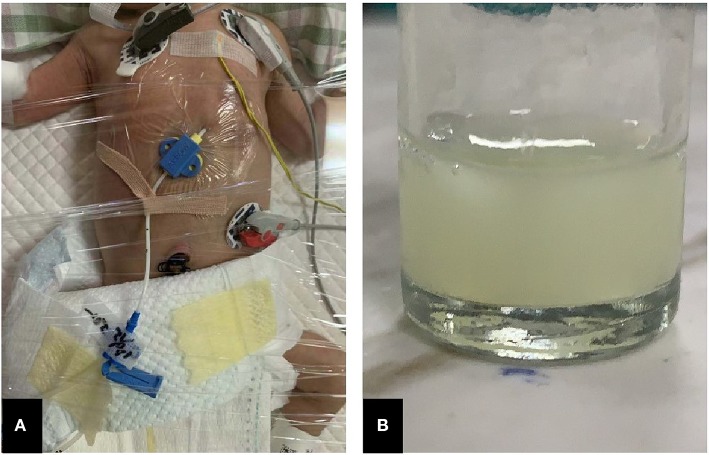
Features of pericardiocentesis and pericardial effusion. **(A)** Pericardiocentesis and pericardial indwelling drainage; **(B)** Milky-white fluid released through pericardial puncture.

The patient was a female infant admitted to our hospital on 6 December 2018 because of a preterm delivery requiring resuscitation efforts for 10 min. The infant was delivered via cesarean section at 30^+1^ weeks' gestation in the Obstetrics Department of our hospital, owing to 48 h premature membrane rupture. The birth weight was 1,370 g, and the Apgar score was 5 at 1 min, and 9 at 5 min after birth. At admission, the patient's heart rate was 140 beats per min, heart sounds were strong, peripheral blood pressure was 50/24 mmHg, and the capillary refill time was 2 s. The patient was intubated with positive airway pressure ventilation, and surfactant was given. Two days after birth, a PICC was inserted via the right basilic vein, and parenteral nutrition was administered. An X-ray showed that the tip of the catheter was located in the superior vena cava. Three days after birth, cardiac B ultrasound showed a patent ductus arteriosus (PDA) with diameter 1.7 mm and ejection fraction of 65 %. On the 8th day of life, the infant suddenly became tachycardic, her skin color became dark, and she developed metabolic acidosis. Heart auscultation was significant for distant heart sounds. Laboratory testing revealed no infections. A repeat chest X-ray showed that the catheter was distorted, and the heart shadow was enlarged with a cardiothoracic ratio of 0.67 (Figure 1A). A cardiac ultrasound showed a large pleural effusion. The ultrasound also showed a right ventricular diameter of 4.4 mm, left ventricular end-diastolic diameter of 13.2 mm, end-diastolic volume of 5.9 ml, end-systolic volume of 2.7 ml, stroke volume of 3.1 ml, and ejection fraction of 53%. The interventricular septum shifted to the left during inspiration and to the right during exhalation (Figure 2). The PICC was removed immediately, and pericardiocentesis was performed, which released 35 ml of milky-white PCE (Figure 3). The patient's condition improved quickly. Her skin color became pink, her heart sounds recovered to normal, and the metabolic acidosis was also corrected. A chest X-ray showed that the heart shadows were reduced, and the cardiothoracic ratio was 0.6 (Figure 1B). A cardiac ultrasound showed that the PCE had completely disappeared. The left ventricular systolic function recovered, with a left ventricular ejection fraction of 69%. The infant was hospitalized for a total of 55 days, and her weight increased to 2,040 g. The infant's condition was good at discharge, and she showed only mild white matter damage. There were no other complications.

## Literature Review

Using “Catheter AND Cardiac Tamponade” or “Catheter AND Pericardial Effusion” as keywords, we searched the PubMed database (for studies with publication dates up to December 2018) and found 13 studies involving PICC-induced PCE/CT. The 13 studies involved 23 cases. We searched the CNKI and Wanfang databases (for studies with publication dates up to December 2018) and did not find any relevant Chinese studies. We then analyzed the clinical data of the 23 cases, as well as the present case, including the insertion pathway, leakage time, catheter tip location, treatment measures, and patient outcomes (Table 1).

**Table 1:** Summary of patient information.

**References**	**Gestational age (weeks)**	**Birth weight(g)**	**Indwelling time(d)**	**Indwelling duration time (d)**	**Indwelling site**	**Location of original catheter tip**	**Whether drift and tip location**	**Clinical manifestations**	**Treatment**	**Outcome**	**Autopsy**
Atmawidjaja et al. (3)	33	1360	Undisclosed	7	Right antecubital vein	Superior vena cava	Pericardial cavity	Reduced blood oxygenation, decreased heart rate, reduced heart sounds	Pericardiocentesis (25 ml)	Dead	Autopsy
Pizzuti et al. (2)	25	620	1	17	Right basilic vein	Junction between Inferior vena cava and right atrium	No drift	Reduced blood oxygenation, decreased heart rate, hypotension, reduced heart sounds	Pericardiocentesis (2 ml)	Improvement	
Darling et al. (4)	24	600	2	16	Undisclosed	Undisclosed	Right atrium	Undisclosed	Undisclosed	Dead	No
	29	1,550	1	2	Undisclosed	Undisclosed	Right atrium	Undisclosed	Undisclosed	Dead	No
	32	1,740	3	2	Undisclosed	Undisclosed	Right atrium	Undisclosed	Undisclosed	Dead	No
	25	890	3	26	Undisclosed	Undisclosed	Right atrium	Undisclosed	Pericardiocentesis (37 ml)	Improvement	
	28	1,130	24	22	Undisclosed	Undisclosed	Right atrium	Undisclosed	Pericardiocentesis (10 ml)	Improvement	
Pezzati et al. (5)	30	1,470	1	1	Undisclosed	Superior vena cava	Right atrium	Cardiac arrest	CPR	Dead	Pericardial effusion 50 ml
	27	1,080	6	5	Undisclosed	Superior vena cava	Right atrium	Cardiac arrest	CPR	Dead	Pericardial effusion 9 ml
	24	610	5	13	Undisclosed	Superior vena cava	Right atrium	Undisclosed	Pericardiocentesis (10 ml)	Improvement	
	30	840	4	8	Undisclosed	Superior vena cava	Right atrium	Undisclosed	Pericardiocentesis (13 ml)	Improvement	
	26	780	10	3	Undisclosed	Superior vena cava	Right atrium	Undisclosed	pericardiocentesis (14 ml)	Improvement	
Nadroo et al. (6)	34	Undisclosed	Undisclosed	4	Undisclosed	Right atrium	Right atrium	Undisclosed	pericardiocentesis (33 ml)	Improvement	
	26	610	5	6	Right antecubital vein	Superior vena cava	Right atrium	Bradycardia, hypotension	CPR	Dead	Pericardial effusion 9.5 ml
Warren et al. (7)	22	580	Undisclosed	18	Right femoral vein	Near the right atrium	Undisclosed	Bradycardia, hypotension	Undisclosed	Dead	Pericardial effusion 8 ml
	41	3,142	Undisclosed	Undisclosed	Undisclosed	Close to right atrium wall	Undisclosed	Deteriorating condition	Undisclosed	Dead	Pericardial effusion 1–2 ml
Cade and Puntis (8)	30	1,240	Undisclosed	12	Right great saphenous vein	Right atrium	Catheter distortion	Increased breathing„ apnea	Pericardiocentesis (23 ml)	Improvement	
Aiken et al. (9)	25	790	Undisclosed	1	Left great saphenous vein	Right atrium	Undisclosed	Hypotension, bradycardia	Pericardiocentesis (8 ml)	Improvement	
Beattie et al. (10)	27	1,040	3	4	Right antecubital vein	Right atrium	Catheter curling	Bradycardia, acidosis, hyperglycemia, poor perfusion	pericardiocentesis (5 ml)	Improvement	
Kulkarni et al. (11)	26	780	10	37	Undisclosed	Superior vena cava	Undisclosed	Undisclosed	Pericardiocentesis (47 ml)	Improvement	
Haass et al. (12)	25	630	3	26	Right basilic vein	Undisclosed	Undisclosed	Undisclosed	Pericardiocentesis (6 ml)	Improvement	Right pleural effusion
Wirrell et al. (13)	27	740	6	32	Right axillary vein	Superior vena cava	Catheter obstruction	Apnea, bradycardia	Pericardiocentesis (23 ml)	Improvement	
Bagtharia et al. (14)	25	Undisclosed	1	1	Left elbow vein	Right atrium	Undisclosed	Bradycardia, hypotension, acidosis	Pericardiocentesis 20 ml	Improvement	

### General Information

The 24 cases included 15 extremely preterm infants at <28 weeks gestation (62.5%), 8 early preterm infants at 28–34 weeks gestation (33.3%), and 1 full-term infant (4.2%). The average birth weight was 1072.4 ± 579.9 g, and the median catheterization time was 3 days. The median PICC indwelling time to developing PCE was 7 days. The catheter insertion pathways included the following: the right antecubital vein (three cases [12.5%]); the right basilic vein (three cases [12.5%]); the right axillary vein (one case [4.2%]); the right great saphenous vein (one case [4.2%]); the right femoral vein (one case [4.2%]); the left great saphenous vein (one case [4.2%]); and the left elbow vein (one case [4.2%]). The other 13 cases (54.2%) had no insertion pathway information reported.

### Catheter Tip Location

The catheter tip locations included the following: drift from the superior vena cava to the right atrium (7 cases [29.2%]); directly in the right atrium (12 cases [50.0%]); directly in the superior vena cava (2 cases [8.3%]); and directly in the junction between the inferior vena cava and the right atrium (1 case [4.2%]). The location of the catheter tip in two cases was not reported (8.3%). There were three cases in which the catheter was distorted or curled (12.5%) and one case in which the catheter was obstructed (4.2%).

### Clinical Manifestations and Outcomes

Cardiac arrest occurred in two patients. One patient had an increased heart rate, one patient developed metabolic acidosis, and one patient had deterioration of the primary disease condition. Other patients had symptoms including bradycardia, apnea, and hypotension. Fifteen patients underwent pericardiocentesis. Five infants died, and pericardial effusions were demonstrated at the time of autopsy. Of the 24 infants, nine died (37.5%), and 15 improved (62.5%). In the infants who underwent pericardiocentesis, only one died, and the mortality rate was 6.25%.

## Discussion and Conclusions

PCE/CT is a rare but severe complication caused by PICCs. PCE/CT has a variety of clinical manifestations, such as dyspnea, bradycardia, and hypotension; however, owing to the lack of specific clinical manifestations, the diagnosis of PCE/CT is difficult, and without immediate treatment, the mortality rate is often high. The currently known risk factors for PICC-induced PCE/CT include catheter tip position, catheter drift, and infusion of hypertonic fluid (15–17).

With respect to the position of the catheter tip, among 19 cases (79.2%) in our analysis, the catheter tip was in the right atrium when the PCE/CT developed. Warren et al. (7) have proposed that when the catheter tip is within the atrium, the myocardial damage and micro-perforation caused by mechanical friction with the atrial wall may be an important cause of PCE/CT. Therefore, when the catheter tip is in the right atrium, close attachment to the atrial wall, into the tricuspid valve, or into the coronary sinus should be avoided; however, PCE/CT cannot be completely prevented even when the catheter tip is outside the right atrium. One potential mechanism that may underlie PCE is that the hypertonic venous nutrient solution containing a fat emulsion can penetrate the atrial wall and damage endothelial cells and the endocardium. Currently, the optimal position of the catheter tip remains controversial. Most studies (5, 18–20) still recommend that the catheter tip be in the vena cava but outside the heart outline (for preterm infants, approximately 1 cm outside of the heart outline, and for full-term infants, approximately 2 cm outside of the heart outline).

In 7 (29.2%) of the 24 infants, the catheter drifted from the superior vena cava to the right atrium. Upper limb movements of newborns can significantly change the PICC position. Nadroo et al. (6) have reported that upper limb movements can change the lengths of the soft tissues of the arms, including the vein length, and additionally cause adduction of the shoulder joint or flexion of the elbow joint, thus squeezing the soft tissues around the catheter and resulting in movement of the catheter toward the centrifugal end. However, if the two movements occur simultaneously, the catheter moves toward the heart. Caution should be taken when infants adduce their arms and flex their elbows simultaneously; because the catheter tip is closest to the atrium, catheter drift is likely to occur. To ensure that upper arm movements do not cause the catheter tip to enter the right atrium, the upper arms should be placed in a special position during X-ray positioning. Beyond catheter drift, catheter distortion is another reason for the occurrence of PCE/CT. The incidence of PICC distortion is approximately 7%; however, the incidence of catheter distortion increases to 55% in infants with PCEs (4). A potential mechanism leading to catheter distortion involves contact between the catheter tip and the atrial wall. The mechanical friction between the catheter tip and the atrial wall can induce inflammation, tissue necrosis, and perforation, thus resulting in PCE/CT.

Owing to a lack of typical clinical symptoms, diagnosis of the disease is difficult in many cases. A PICC-induced PCE can lead to sudden CT, which has a 75% mortality rate; however, in patients undergoing pericardiocentesis, the mortality decreases to 8% (1), which is close to the mortality (6.25%) among the 24 cases examined. Some researchers have suggested that if infants with PICC have a sudden instability in cardiopulmonary function that cannot be explained by other reasons, emergency pericardiocentesis and drainage should be considered (14). In addition, direct drainage with a PICC can be performed with sudden CT, although the approach may not be successful. Therefore, although PICC drainage is an option, heart ultrasound or pericardiocentesis cannot be delayed if PICC drainage is unsuccessful. In addition, ECG examination for pericardial effusion in premature infants has some limitations. With adult acute pericardial tamponade, ECG can show non-specific ST-T changes, with P wave, QRS wave, and T wave alternation. Total heart electrical alternation is the characteristic ECG of pericardial tamponade. However, ECG examination for pericardial effusion in premature infants has some limitations. Because of the thin chest wall in newborns, especially in premature infants, and the elevated diaphragm, these physiological and anatomical characteristics affect the amplitude of the P-QRS wave. Therefore, the ECG characteristics differ from those in adults and older children, which affects clinical interpretation. Besides, ECG examination of premature infants must be performed under sufficient sedation, which increases the difficulty of examination. Therefore, we did not perform ECG examination in this patient.

The position of the PICC may be changed according to weight changes in VLBW infants after birth, the need for repeated tube flushing, and excessive limb movement on the PICC puncture side. After our experience with this patient, we try to avoid large limb movements on the puncture side during daily procedures, and we perform chest X-ray examination to ensure a clear PICC route and tip location at least once a week. If the route is abnormal or the tip of the catheter drifts, the catheter should be adjusted quickly to avoid complications. In some infants developing PCE, a PICC can still be used normally if the catheter is retracted out of the right atrium and the PCE does not recur, thus suggesting that the PICC need not be removed in all infants; however, close monitoring is required (3). In our case, because the patient was in an extremely unstable condition at that time, we were unsure whether leakage, distortion, and other factors were present regarding the PICC, in addition to the abnormal PICC position. Therefore, we removed the PICC and replaced it with peripheral venous access.

Leakage of fluid into the pericardial cavity, as a result of myocardial perforation caused directly by a catheter tip or tissue necrosis caused by hypertonic fluid, can lead to CT and sudden death. Although the incidence of this condition is low, the mortality rate is extremely high. Because of the lack of specific clinical manifestations and low incidence, medical staff often ignore this condition and delay diagnosis and treatment (21). For all neonates with a PICC, if unexplainable and refractory dyspnea, apnea, abnormal heart rate and blood pressure, metabolic acidosis, or deterioration of the original disease conditions are present, caution should be taken regarding PICC-induced PCE/CT. Immediate pericardiocentesis can significantly decrease the mortality rate of the disease. In summary, awareness of the possibility of PICC-induced PCE/CT in the target population, timely examination, diagnosis, and pericardiocentesis are critical for reducing mortality and improving the prognosis of the disease.

## Data Availability Statement

All data generated or analyzed during this study are included in this published article and its supplementary information files.

## Ethics Statement

Written informed consent was obtained from the legal guardian/next of kin of the participant for the publication of any potentially identifiable images or data included in this article.

## Author Contributions

AH conceptualized and designed the case report, reviewed and revised the manuscript, and approvedthe final manuscript as submitted. JF developed initial draft, reviewed and revised the manuscript, and approved the final manuscript as submitted.

## Conflict of Interest

The authors declare that the research was conducted in the absence of any commercial or financial relationships that could be construed as a potential conflict of interest.

## Abbreviations

CNKI: China National Knowledge Infrastructure.
